# Broader Screening for Youth-Onset Type 2 Diabetes—We Just Are Not There Yet

**DOI:** 10.1001/jamanetworkopen.2022.20540

**Published:** 2022-09-01

**Authors:** Amy S. Shah, Kristen J. Nadeau, Megan M. Kelsey

**Affiliations:** Division of Pediatric Endocrinology, Cincinnati Children's Hospital Medical Center, The University of Cincinnati, Cincinnati, Ohio; JAMA Network Open, Chicago, Illinois; Section of Endocrinology, Department of Pediatrics, Children's Hospital Colorado, University of Colorado School of Medicine, Aurora; Section of Endocrinology, Department of Pediatrics, Children's Hospital Colorado, University of Colorado School of Medicine, Aurora

Prior to the 1990s, almost all childhood-onset diabetes could be attributed to insulin-dependent type 1 diabetes. However, as the prevalence of obesity has increased across the US, so has the number of children and adolescents diagnosed with youth-onset type 2 diabetes. Recent US data suggest that the prevalence of type 2 diabetes in youth has increased 2-fold between 2001 and 2017.^1^ The Centers for Disease Control and Prevention reported that between 2005 and 2016, 1 in 5 teens in the US had prediabetes,^2^ defined as blood glucose levels that are higher than reference range but do not meet criteria for diabetes. A further spike in the incidence of youth-onset type 2 diabetes has occurred during the COVID-19 pandemic.^3^

Given the increasing rates of prediabetes and youth-onset type 2 diabetes and associated comorbidity, the US Preventive Services Task Force (USPSTF) assessed whether there is evidence to support screening asymptomatic children and adolescents for type 2 diabetes.^4,5^ The USPSTF concluded that there is "insufficient evidence to assess the balance of benefits and harms of screening for prediabetes and type 2 diabetes in youth" (I statement).^4,5^ This was owing to a dearth of studies addressing the benefits or harms of screening for prediabetes and type 2 diabetes associated with health outcomes in the pediatric age group.^4,5^

The statement recommending neither for nor against screening for prediabetes appears to be well-founded.^4,5^ Not all youth with obesity or prediabetes progress to type 2 diabetes. A Cochrane Database Systematic Review^6^ found that the pooled cumulative incidence of type 2 diabetes in youth with prediabetes after a follow-up of 1 to 10 years was 1% to 56%. Moreover, rates of reversion to glycemia within reference range were between 45% and 81%.^6^ There are also limitations in the screening methods used in youth. A study by Kelsey et al^7^ found that 2.2% of adolescents with weight within reference range had elevated hemoglobin A_1c_ levels and fasting glucose concentrations within the prediabetes range. This study used the prediabetes definition recommended by American Diabetes Association, which is based on adult data demonstrating cutpoints that project progression to diabetes.^8^ Similar data do not exist in youth.

Even if we could more clearly define prediabetes in youth, there are currently no demonstrated interventions that prevent progression from prediabetes to youth-onset type 2 diabetes and no evidence that early identification of prediabetes in youth carries any long-term benefit. Studies that better determine how prediabetes should be defined in youth, as well as studies that identify better targets for prevention and, thus, more effective prevention strategies, are needed. In fact, when directly compared with adults in the Restoring Insulin Secretion (RISE) study,^9^ youth with prediabetes and type 2 diabetes were more insulin resistant, had higher insulin secretion at baseline, and had faster rates of pancreatic β-cell decline over time. Additionally, unlike adults, treatment with metformin or insulin failed to prevent this β-cell decline in youth with prediabetes or type 2 diabetes. Therefore, the National Institutes of Health is now developing a large consortium with a first step goal to better refine which youth are at highest risk to develop type 2 diabetes.^10^ It is likely that the findings of this work, across 15 clinical centers in the US once selected, may inform future guidance on whom to screen and the benefits or harms associated with early prediabetes detection in in the pediatric age group.

The question of the value in screening for type 2 diabetes in youth considered to be at high risk is a little more challenging. Compared with the prevalence of obesity in adolescents, at approximately 20%, the prevalence of type 2 diabetes in adolescents is low, at approximately 0.67 per 1000 adolescents.^1^ Furthermore, studies show that screening for type 2 diabetes in youth with high risk is of low yield: in the US, screening for type 2 diabetes based on fasting and postchallenge blood glucose levels in adolescents with high risk identified type 2 diabetes in less than 1% of adolescents.^11^

However, once type 2 diabetes in youth is identified, it is associated with more rapid progression of β-cell decline, as demonstrated in the RISE study, as well as earlier onset of comorbidities and complications than typically seen in adults. The Treatment Options for Type 2 Diabetes in Adolescents and Youth (TODAY) study^12^ found significant early onset complications, with 60.1% of youth studied having at least 1 microvascular complication by age 26 years. While it is true, as the USPSTF recommendation statement and evidence review^4,5^ point out, that the treatments in the TODAY study failed to prevent rapid β-cell decline and early complications, there was no placebo comparison, nor was there in the RISE study. Certainly, the alternative of late identification and no treatment of type 2 diabetes in youth is unlikely to be associated with better outcomes. In fact, there is direct evidence from the TODAY study^13^ that achieving a lower hemoglobin A_1c_ (<6.3%; to convert to proportion of total hemoglobin, multiply by 0.01) early in the diagnosis of diabetes was associated with better outcomes. Thus, while evidence for the benefit of making an early diagnosis of prediabetes in youth is limited, there may be a direct benefit in presymptomatic identification and treatment of type 2 diabetes in this age group. Moreover, glucagon-like peptide receptor agonists, which are now approved in youth, and sodium–glucose cotransporter 2 inhibitors, which are under study for approval in youth, are associated with weight loss benefits and unique long-term cardiovascular and kidney health benefits in adults.^14^ Additionally, outcomes from youth with type 2 diabetes in the Teen Longitudinal Assessment of Bariatric Surgery study^15^ suggest that bariatric surgical treatment offers promise and that outcomes may even be better if the treatment is performed before adulthood.

The American Diabetes Association recommends screening for prediabetes and type 2 diabetes based on the youth's risk profile.^16^ For children who are aged at least 10 years or have started puberty, screening is recommended if body mass index is greater than the 85th percentile and at least 1 of the following conditions is present: (1) there is a family history of type 2 diabetes in a first- or second-degree relative; (2) the youth self-reports belonging to a minority race or ethnicity group (eg, American Indian, Asian, Black, Hispanic, Middle Eastern, or Pacific Islander); (3) there are signs of insulin resistance (eg, acanthosis nigricans, hypertension, dyslipidemia, or polycystic ovary syndrome) or the youth was born with a low or high birth weight; or (4) if there is a maternal history of type 2 diabetes or gestational diabetes during the child's gestation.^16^ These recommendations are based on the current evidence surrounding risk factors in youth in hopes of minimizing unnecessary testing. While this screening approach will certainly result in identification of dysglycemia of unknown significance, we argue that clinicians need some way to detect true diabetes before it progresses to a point at which available treatments are ineffective at preventing complications.
